# Poverty after Birth: How Mothers Experience and Navigate U.S. Safety Net Programs to Address Family Needs

**DOI:** 10.1007/s10826-022-02322-0

**Published:** 2022-05-06

**Authors:** Maria Marti-Castaner, Tonya Pavlenko, Ruby Engel, Karen Sanchez, Allyson E. Crawford, Jeanne Brooks-Gunn, Christopher Wimer

**Affiliations:** 1 Copenhagen University, Department of Public Health, Section of Health Services Research, Copenhagen, Denmark; 2 The New School for Social Research, New York, NY USA; 3 Columbia University, Center on Poverty and Social Policy, New York, NY USA; 4 Former CEO, Room To Grow and Founder, evolutionforward, New York, NY USA; 5 Teachers College, Columbia University, New York, NY USA

**Keywords:** Poverty, Maternal health, Qualitative research, Homelessness, Housing, Early childcare

## Abstract

Although pregnancy and the first year of life are sensitive windows for child development, we know very little about the lived experiences of mothers living in poverty or near poverty during the perinatal period; specifically, how they perceive and use public resources to support themselves and their newborn. In this qualitative study, we explore how predominantly Black and Latinx mothers with infants living in or near poverty and engaged in public assistance manage to meet their family’s needs with available resources from safety net programs and social supports. We conducted 20 qualitative interviews with mothers living in (85%) or near poverty in New York City (NYC). All participants (mean age = 24) had an 11-month-old infant at the time of the interview. Using thematic analysis, we identified five main themes reflecting how mothers experience and navigate living with very low incomes while engaging in public assistance programs: (1) experiencing cascading effects of hardships during pregnancy, (2) relying on food assistance and informal supports amid scarcity, (3) waiting for limited affordable housing: ‘life on hold’, (4) finding pathways towards stability after the baby’s birth, (5) making it work: efforts to look forward. Results describe how the current focus on “work first” of existing federal and state policies adds a layer of stress and burden on the lives of single mothers experiencing low incomes and entangled hardships during pregnancy and after birth. We document how mothers experience coverage gaps and implementation challenges navigating the patchwork of public assistance programs, yet how the support of flexible caseworkers accessing, using, and coordinating assistance has the potential to help mothers plan for longer-term goals.

## Highlights

- Mothers with low incomes experience multiple needs regarding income, housing, and childcare despite existing safety net programs.
- Mothers find strength through their relationships with their children and the support encountered and mobilized from the community and family.
- Safety net programs may not always be sufficient for mothers with low incomes in the first year after giving birth.
- Safety net programs must review coverage gaps, implementation challenges, and work requirements for mothers of infants.

The United States’ engrained lines of social inequality are reflected in the experience of poverty, with women and children—especially women of color—facing poverty at disproportionately high rates. In 2018, 12.9% of women and 16.2% of children lived in poverty in the United States; those numbers were nearly double among Black and Latinx women and children (Semega et al., 2019). For families living in poverty or with low incomes (below twice the federal poverty line), particularly female-headed households, the birth of a baby has often been associated with increased economic strain and reduced financial mobility out of poverty (Noelke et al., 2019; Stanczyk, 2016). Although pregnancy and the first year of life are well-established sensitive windows for child development (Duncan et al., 2012; Roos et al., 2019), there is still sparse research about the lived experiences of mothers of color experiencing poverty during that time and. specifically, how they perceive and navigate resources to support themselves and their infants. This study explores how mothers living in New York City (NYC) in (85%) or near poverty and using public assistance programs (100%) manage to meet (or not) their families’ needs while mobilizing social supports and navigating the patchwork of public assistance programs during pregnancy and the first year of their child’s life.

## The Importance of Reducing Poverty during Early Childhood

The impact of poverty on pregnancy and early childhood development is well-documented (Chaudry & Wimer, 2016; Duncan & Brooks-Gunn, 1997; Duncan et al., 1998; Yoshikawa et al., 2012). In the time surrounding pregnancy and birth, all women are at increased risk of experiencing at least one hardship, such as financial strain, job loss, separation from their partners, domestic violence, or homelessness but this is particularly accentuated for women who are already living with low incomes (Ahmadabadi et al., 2020; Braveman et al., 2010; Katz et al., 2018). Poverty has also been linked to increased maternal mortality rates (Gingrey, 2020; Singh, 2021), particularly among Black women, as well as deleterious health outcomes for mothers, including higher rates of chronic hypertension, diabetes and obesity – conditions that are associated with an increased risk for birth complications and job loss during pregnancy (Bombard et al., 2012; Tanya Nagahawatte & Goldenberg, 2008). The chronic stress associated with poverty can also negatively affect mothers’ mental health (Wickham et al., 2017). Subsequently, children who are born into families experiencing poverty disproportionately face adversity (Frank et al., 2010), including higher frequencies of trauma (Merrick et al., 2018), food insecurity (Coleman-Jensen et al., 2018), substandard health care (Jetelina et al., 2018; Kushel et al., 2006), and housing insecurity (Sandel et al., 2016). It is thus during this critical window when the safety net should be most effective at preventing the pervasive effects of poverty and protecting women with already low incomes from falling deeper into poverty and distress. This is especially true for single-mothers, who are likely to face increased financial burdens and tend to have fewer material resources and poorer mental health than partnered mothers (Waldfogel, Craigie, & Brooks-Gunn).

## Current Safety Net Programs in the US During Pregnancy and Early Childhood

Since 1996, with the introduction of the Personal Responsibility and Work Opportunity Reconciliation Act (PRWORA), the American social safety net has placed a strong focus on work-based assistance that seeks to increase labour force participation, particularly among female heads of households (Curran et al., 2021). In this welfare reform lies the recent origins of the current social safety net for children and families in the US, which is characterized by an increase in tax credits for working families, a large reduction on cash assistance for the poorest, work requirements, and an absence of universality (Shaefer et al., 2020). Below we provide a summary of current means-tested anti-poverty programs relevant to pregnant women and their children that focus on nutrition, healthcare, income support, childcare and housing (see supplemental material Table s1).

WIC (Special Supplemental Nutrition Programs for Women, Infant, and Children) and SNAP (Supplemental Nutrition Assistance Program) are the main means-tested federal nutrition programs that provide near-cash transfers to buy food for families with low incomes and one of the few that do not have work requirements for women taking care of a child under six. For healthcare, some families with low incomes participate in Medicaid, a joint federal and state program, along with the Children’s Health Insurance Program (CHIP), which provides health insurance coverage to pregnant women and children with low incomes.

Another major safety net mechanism for low-income families is income support. Tax policies, such as the Earned Income Tax Credit (EITC) and the Child Tax Credit (CTC), provide once-a-year cash income assistance, with both credits historically contingent on employment, excluding many of the most disadvantaged families whose earnings are not high enough to qualify for the full credits (Collyer et al., 2020). In 2019, one-third of all children did not receive the CTC, with the most affected being children in single-parent households and children of color (Collyer et al., 2020). In addition to tax policies, pregnant women and parents of children under 18 who experience poverty are eligible to receive cash assistance through the TANF (Temporary Assistance for Needy Families) program. TANF rules and benefits vary from state to state, yet since PRWORA, cash benefits became tied to employment, requiring recipients to work or be actively looking for a job, except for women caring for a child below three months of age (Giannarelli et al., 2017). Currently, almost 90% of TANF recipients are single mothers. However, TANF now covers relatively few mothers. Today, approximately 30% of eligible families receive TANF, compared to 80% in the late 1980s (Tach & Edin, 2017). Such decrease is explained by its time-limited nature, stringent work requirements, and many states’ attempts to keep mothers off the program and use TANF funds for various non-cash assistance purposes (Shaefer et al., 2020).

Subsidized childcare services are available to families with low incomes through vouchers, which cover a portion of the costs, or slots in programs offered by state-certified providers. While there have been groundbreaking efforts to ensure universal early education for three and four-year-olds, slow progress has been made on increasing the numbers of high-quality care for infants and toddlers (Love et al., 2013). Childcare assistance does not reach all eligible families with low incomes. In 2017, only 22 percent of all eligible children under state rules were receiving childcare subsidies (ASPE, 2020).

Housing assistance via vouchers to rent housing in the private market (Section 8) or by renting directly in public housing units is also available to families with low and moderate incomes (Kingsley, 2017). However, only one in four income-eligible households with children receive public housing assistance due to limited funds. Voucher programs have a long waiting list and families often find themselves waiting for up to 10 years to receive a voucher (Housing & Development, 2000).

Taken together, the U.S. maintains a number of discrete and only sometimes-related programs to meet the needs of families experiencing periods of low incomes. These programs target different needs and have different eligibility requirements. Some scholars have thus described the U.S. safety net as a “patchwork” (Bitler et al., 2021; Fishback et al., 2010), one that potentially leaves holes when it comes to providing for mothers and infants, a time when robust and coordinated assistance may be needed most.

## Benefits and Pitfalls of Safety Net Programs: A Need for Mothers Perspectives

Large scale studies on the impact of safety net programs have demonstrated poverty reduction (Pac et al., 2017; Shapiro & Trisi, 2017) and improved maternal (Evans & Garthwaite, 2014) and child outcomes (Bronchetti et al., 2019). Among means-tested programs, SNAP and tax credits have the largest impact on overall poverty rates, especially among children (Carlson & Keith-Jennings, 2018; Fox et al., 2020). Housing assistance and TANF, have also shown impacts on poverty rates, but to a smaller degree (Chaudry et al., 2017). In addition, access to subsidized early education, especially of high quality, has been shown to alleviate some of the consequences of poverty by enhancing children’s cognitive and social development, and allowing parents to participate in the labor force (Chaudry & Sandstrom, 2020; Hotz & Wiswall, 2019).

However, even though the full safety net has improved the economic well-being of families over the past 50 years (Chaudry et al., 2017; Fox et al., 2015), deep poverty (cash income below 50 percent of its poverty threshold) is still a pressing problem (Fox et al., 2015). Since the 1990s, deep poverty has doubled, and currently, about two million children, or 2.7 percent of all children, live in deep poverty, with a higher percentage for black children (4.5 percent) and single-mother households (5.3 percent) (Fox, 2019). While there is evidence that the welfare reform has benefited working families with low incomes by making more eligible to tax benefits and food assistance, recent research argues that it may be increasing the number of low-income households that fall through the cracks of the work-based safety net and struggle to move out of poverty (Shaefer et al., 2015).

Scholars have pointed to a number of shortcomings of existing programs, such as coverage gaps, implementation challenges, burdensome application processes, and strict work requirements that overall may limit access, use and impact of public assistance among the most vulnerable (Danziger, 2010). For example, SNAP, although an essential financial resource to pay for food, rarely sustains families with very low incomes throughout an entire month (Edin et al., 2013; Nieves et al., 2021). Research has also pointed to several barriers to using housing vouchers, such as landlords not wanting to rent apartments to voucher holders, the unacceptability of available housing, and unaffordability (Livingstone & Herman, 2017; Teater, 2011). Indeed, on average 30% of individuals who received Section 8 vouchers do not succeed using them to lease units (Buron, 2001). In addition, a small but growing body of qualitative research has highlighted how the stricter welfare regulations that focus on “work first”, together with unmeet childcare needs and unpredictable schedules at low-wage jobs has contributed to work-family conflicts for single mothers with low incomes, impeding upward mobility (Danziger, 2010; Freeman & Dodson, 2020; Freeman, 2017). Research has documented how mothers that need to turn to public assistance may experience difficulty applying to and retaining government benefits, particularly child care and cash assistance, due to burdensome requirements or misinformation, while simultaneously finding low-wage jobs that allow them to meet their family responsibilities with limited supports at home (Hays, 2004; Pearson, 2007). Freeman and Dodson (2020) described the conflicts and despair that low-income mothers experienced when trying to meet work, parenting, and welfare demands- what they named as the ‘*triple role overload’*. Research has also demonstrated that the current safety net may inadvertently push recipients, particularly single mothers of color, to find low-paid jobs instead of pursuing higher education, reducing their chances of moving out of poverty in the long term (Burke et al., 2021; Pearson, 2007; Safawi & Floyd, 2020).

This body of work has brought valuable insights into the complex challenges that mothers living with low incomes in the U.S. face and how work-family conflicts have been accentuated by work requirements tied to public assistance. However, it has focused mainly on mothers of preschool and school-aged children, and has paid little attention to how issues of limited affordable housing intersect with work-family conflicts. For example, Freeman and Dodson (2020) study focused mainly on mothers that were already residing in public housing. In addition, the experiences of mothers in more vulnerable positions, for example experiencing homelessness, have been underrepresented. A look into the particular lived experiences of women living in poverty during pregnancy and childbirth, a sensitive period in which stress, maternal mental health, and financial resources can have large impacts on infant well-being, is much needed. What leads women to need public assistance and shelter during pregnancy? How do they access, use, and navigate the “patchwork” of public assistance after giving birth to meet their and their infants’ needs? And how do they experience the benefits and pitfalls of a complex safety net that in the last two decades has increased its focus on labour market attachment, while they care for small infants and try to move out of poverty? We aimed to explore these questions by delving into the lived experiences of predominantly Black and Latinx mothers for the most part living in poverty connected to public assistance programs and living in NYC during pregnancy and the first year of their infant’s life. Specifically, we sought to understand the complexity of women’s experiences and needs during this sensitive period and how they navigate public assistance to meet (or not) their essential material and psychosocial needs. Further, we explored how women’s use of their social networks and supports intersect with their need for and use of public assistance and what role housing instability, particularly for women in and out of shelters, played. Programs and policies that aim to assist pregnant women and infants coming from economically poor households stand to benefit from more nuanced qualitative data exploring when, why, and how assistance is utilized, and where it may fall short during this sensitive period.

In this study, we use the term public assistance to refer to public (social) government assistance including food assistance, housing assistance, childcare vouchers, and cash assistance. Although Medicaid is one of the largest anti-poverty-reduction policies in the U.S., we focus on policies intended to help mothers meet their immediate and routine economic needs of providing food, housing, childcare, and purchasing goods and services for their new baby. In addition, all women in the study qualified for Medicaid during their pregnancy and none of them reported healthcare expenses during their pregnancy and birth, thus leading us to conclude that they had health insurance that met their immediate healthcare needs following the birth, at least in the short term. In this study, we refer to participants as ‘women’ and ‘mothers’ although at the time of research we did not ask for gender identity. We acknowledge this as a limitation and recognize that perhaps not all participants identify as women.

## Methods

### Study Context

The study took place in New York City (NYC), where one in five children live in poverty (an annual income below $25,926 for a family of two adults and two children as of 2019) (Shin et al., 2018) New York State is one of the top benefit spenders. It has implemented family-focused programs such as the state-funded family paid leave or EarlyLearn NYC that offers free or low-cost childcare (from 6 weeks of age) (see supplemental materials). In addition, New York City’s shelter system for homeless families is consistently recognized as the most comprehensive in the nation. However, NYC still faces childcare and housing challenges that affect families with low incomes. For example, in 2019, Administration for Children’s Services (ACS), which runs EarlyLearn NYC, served around 23,000 infants and toddlers, representing only 14% of children under three in low-income households. In addition, the limited affordable housing and housing support, coupled with employment instability, evictions, and domestic violence, continues to push many families with young children into homelessness. From 2011 to 2018, the number of families with children in NYC shelters grew by 55% (Institute for Children, Poverty & Homelessness, 2019). Family-certified shelters in NYC provide a range of services, which may include access to daily nutritional meals, supervision, assessment services, permanent housing preparation, information and referral services, access to health services and childcare. However, shelters vary in the amount and quality of services provided. For example, a recent report showed that within certified shelters in NYC, 37% did not offer meals (Office of the New York State Controller, 2018). Thus, the support offered at the shelter in which families are placed may have important consequences for both parents and children.

### Study Setting

This qualitative study was part of an ongoing randomized clinical evaluation (RCT) of Room to Grow, a program provisioning parents living in low-income households with social and material support (MASKED FOR REVIEW). Room to Grow offers one-on-one visits with a social work clinician every three months, from the end of pregnancy to the child’s third birthday. The program’s overall goal is to ensure that parents have the resources they need by offering structured parent coaching, material goods (books, toys, clothing, and baby equipment) and community referrals. The RCT follows 317 mothers and babies living in NYC. Roughly half of the enrolled subjects are in the treatment group, receiving services from the Room to Grow program, and the other half are in the control group, who are not. Mothers participating in the evaluation participate in a baseline survey in their third trimester of pregnancy, before their first visit with the program. The next follow-up survey is conducted when babies reach 10.5 months of age. At the time of this qualitative study, treatment moms had met with their social work clinicians a total maximum of four times. The qualitative study was conducted as part of the 10.5-month evaluation; both control and treatment group participants were included in the qualitative study.

In the larger evaluation of Room to Grow that participants were recruited from, 25% of mothers were living in a homeless shelter toward the end of their pregnancy, and 17% were in a shelter when children were 10 months. Near the end of the pregnancy, roughly 70% of the sample was living in poverty and the rest had incomes between 100 and 200% of the official poverty line. Nearly 90% of the sample was Black or Latinx (MASKED FOR REVIEW).

### Participants and Recruitment

We completed 20 semi-structured interviews between July and December of 2018 with mothers of 10.5-month-old infants, aged 17 to 39 years. Participants participating in the RCT were invited to participate in semi-structured interviews on a rolling basis when their children were 10.5 months. All mothers participating in the larger evaluation were eligible. We aimed to interview the same number of participants in both treatment and control groups. Throughout the recruitment process, two mothers refused to participate and six were hard to reach and after several failed attempts to contact them they were excluded from the list of potential participants. Recruitment ended when new themes and sub-themes did not emerge and existing themes were replicated indicating a level of data saturation (Morse, 1995). Additional interviews could have added nuances to our analysis or may have uncovered more exceptions to the data. Nonetheless, the rich data from 20 participants was sufficient to respond to the aims of this exploratory in-depth investigation.

Participants were approached individually by one of our team members, who explained the nature of the study. Research staff obtained informed consent prior to starting the interview. Subjects were made aware that participation was voluntary, and would not impact Room to Grow services or future involvement in the RCT study. Researchers discussed the potential risks and benefits of participation and ensured that participants were aware of their rights to decline any question and withdraw from the study. Participants who agreed to participate had the opportunity to ask questions and gave informed consent. The study was reviewed and approved by the Columbia University Institutional Review Board. The procedures adhered to the tenets of the Declaration of Helsinki.

### Interview Protocols

Socio-demographic data were collected as part of the larger RCT evaluation and was used to characterize the sample. We developed a semi-structured interview guide following a chronological approach, starting with questions about participants’ life before pregnancy, during pregnancy and birth, and the first year of their child’s life. Questions were designed to capture mothers’ experiences with motherhood, parenting, finances, health, well-being, and psychosocial support, and to understand participants’ experiences using public programs as they sought to meet their needs (supplemental material). The semi-structured nature of the interview guide ensured that planned topics were discussed while allowing for the development of unforeseen themes and the discussion of issues relevant to the participant. Interviews were audiotaped and conducted in English (17), and Spanish (3) by native speakers trained by the lead author, a clinical psychologist, who co-conducted the first four interviews. All interviews were conducted in a private space and lasted 60 min on average; all were transcribed and translated to English. After each interview, researchers wrote a summary with field notes and debriefed the interview with the team.

### Data Analysis

We used thematic analysis to code and analyze all interviews (Clarke et al., 2015) using NVivo12 (International, Q (2012)). We followed five stages: familiarization with data, generation of initial codes, grouping codes into categories, grouping categories into themes and sub-themes, and defining and naming themes (Clarke et al., 2015). The analysis was performed iteratively following an inductive approach. First, three coders reviewed 25% of the transcripts several times to familiarize themselves with the content and emergent themes, independently creating an initial list of codes and categories. Coders then used consensus to develop and refine the codebook until agreement was achieved for all major codes and categories. Then, each coder analyzed one-third of the interviews using the codebook. If new codes and categories emerged during the coding process, these were discussed, incorporated in the coding scheme, and used to recode previously coded interviews. We then grouped codes into sub-themes and themes related to the study aim (see supplemental materials). To ensure data quality interpretation of findings were discussed and compared until agreement on the main themes and sub-themes was met following an iterative and reflexive process (Patton, 1999). For the codes under each sub-theme, the first author conducted cross-case analyses, with each respondent representing a case, to identify differences and similarities across participants and then explored patterns within and across groups (e.g., shelter vs. no shelter). This approach allowed us to organize and find meaningful patterns across the data (Patton, 1999). In addition, after coding all the interviews, the first author reviewed the data for evidence contradictory to identified themes and sub-themes, ensuring that the complexity of participants’ narratives was represented in the results (Creswell and Miller, 2000). In the following section, we describe the main themes illustrated by rich quotes using invented initials to identify participants while keeping their anonymity.

## Results

Table 1 provides sample characteristics. The majority of participants (85%) were living in poverty, with the remaining participants in near poverty just above the poverty line. Overall, half of the participants were first-time mothers. Most had completed at least a high school degree or equivalent (*N* = 14). Nine out of twenty were living in shelters at the time of the interview. Five out of 20 had been victims of domestic violence since they became pregnant. Only two mothers were living with their partners at the time of the interview. Nine out of twenty had their children attending childcare. All were receiving at least two forms of public assistance supports. Nine had recently received housing assistance, although not all were using it yet. A greater number of participants living in shelters received public assistance, demonstrating the vulnerability of this group.

**Table 1:** Sample characteristics at the time of interview (10-month survey)

	Lives in shelter/temporary housing (*n* = 9)		Lives in an apartment/room (*n* = 11)		Full sample (*n* = 20)	
	*n*	%	*n*	%	*n*	%
Age (median, range)	24	20–36	24	17–39	24	17–39
Race/Ethnicity						
Black	5	55.6%	4	36.4%	9	45.0%
Hispanic	3	33.3%	6	54.5%	9	45.0%
Asian	–	–	1	9.1%	1	5.0%
Other	1	11.1%	–	–	1	5.0%
Primary language						
English	8	88.9%	9	81.8%	17	85.0%
Spanish	1	11.1%	2	18.2%	3	15.0%
Education						
Some high school	2	22.2%	3	27.3%	5	25.0%
HS diploma or GED	3	33.3%	4	36.4%	7	35.0%
Vocational or technical school	–	–	1	9.1%	1	5.0%
Some college or 2-year degree	4	44.4%	3	27.3%	7	35.0%
Income-to-needs ratio^a^						
=<1:poor	9	100%	8	72,7%	17	85.0%
=<2:low income	–	–	3	27,3%	3	15.0%
Employment						
Not working outside the home	3	33.3%	4	36.4%	7	35.0%
Looking for work	4	44.4%	–	–	4	20.0%
Full-time job	1	11.1%	1	9.1%	2	10.0%
Part-time job			5	45.5%	5	25.0%
Freelance	1	11.1%	1	9.1%	2	10.0%
Number of children						
1	3	33.3%	7	63.6%	10	50.0%
2 or more	6	66.6%	4	35.4%	10	30.0%
No relationship with child’s father	5	55.6%	1	9.1%	6	30.0%
Living conditions						
Living with extended family/ pays rents	–	–	4	36.4%	4	20.0%
Living with extended family/ does not pay rent	–	–	3	27.3%	3	15.0%
Rent a room from not family friends	–	–	1	9.1%	1	5.0%
Rent own apartment			3	27.3%	3	15.0%
Active Mental health services	6	66.7%	3	27.3%	9	45.0%
Public Assistance						
SNAP	8	88.9%	8	72.7%	16	80.0%
WIC	8	88.9%	11	100.0%	19	95.0%
Public cash/welfare/or disability incomeb^b^	7	77.8%	4	36.4%	11	55.0%
Housing assistance	6	66.7%	3	27.3%	9	45.0%
Childcare voucher	3	33.3%	3	27.3%	6	30.0%

Note. ^a^Income-to-needs ratio=self-reported total family income divided by the federal poverty threshold given the size of the family. When = <1 indicates that the family lives at or below the poverty threshold; =<2 indicates that the family lives between 100–200% of the poverty line. ^b^They have received it at some point before the interview and some continued to receive it (*n* = 9)

Across all interviews, we identified five global themes that describe the experiences and needs that women encounter from pregnancy to the baby’s almost first birthday: 1) experiencing cascading effects of hardships during pregnancy, 2) relying on food assistance and informal supports amid scarcity, 3) waiting for limited affordable housing: ‘life on hold’, 4) finding pathways towards stability after the baby’s birth, 5) making it work: efforts to look forward. Within themes, we identified patterns among subgroups of women if and when differences were seen.

### Experiencing Cascading Effects of Hardships During Pregnancy

The circumstances surrounding the time of pregnancy varied for mothers in the study, as shown in Table 1. However, all women described how their pregnancies were unplanned. When women became pregnant, they were living day-to-day and reported limited possibilities for saving money for their future babies. Most women described how their pregnancies accentuated job and housing instability, “*pushing*’ them to need more support from their families and the safety net. Women described four main drivers of instability during pregnancy: job loss and lack of unemployment benefits, health problems, interpersonal conflicts, and/or unresponsive fathers. These stressors were often interconnected and led women to experience extremely challenging situations - both psychologically and financially. As L.L, who already had three children, explained:

“It changed so much (when she became pregnant) because the father did not take responsibility. Psychologically, emotionally, financially. Everything really changed. I also lost my job because of my diabetes. I ended up with the emotional, financial, social burden. The entire world fell on my shoulders because that’s what I got. I was practically alone here”.

These hardships were intertwined and interdependent, one often exacerbating the other. This was especially true for women that suffered from abusive relationships, for which the stress and trauma of experiencing verbal and in some cases physical abuse compounded health complications during pregnancy, and made them more vulnerable to losing their jobs. A.A. described these hardships:

“I found out I was pregnant, and things just went extremely downhill with my husband. I got really sick ‘cause I have polycystic ovarian syndrome. I was bleeding. I had to go on bed rest. I ended up missing a lot of work. In between the drama with my husband and then bedrest and all of that I had to resign.”

A.A. exemplified the cascading effects that interpersonal conflicts with the child’s father and insecure jobs created during pregnancy. In low paid and temporary jobs, having health complications related to pregnancy was often a reason for being fired or forced to quit their jobs. All but one of the women that were working did not keep their jobs after giving birth or were fired due to health-related problems. L.L also lost her job. She was fired due to complications from her diabetes. “*I was working in housekeeping, but my health was not good because I had diabetes. What the agency should have done was—because I investigated later—to put me on leave and pay me, but that’s not what they did.”* While unemployment benefits would have allowed her and other participants that were working to stay afloat, she did not receive them, despite being eligible as she later found out. Most participants were not eligible to receive sick leave given their unstable employment (short-term contracts, freelance, irregular work) and the few that were, struggled to access it. When L.L. found out that she should have received a paid leave, she went back to speak with her employer to claim it. However, the delay resulted in a temporal loss of income, which brought a lot of stress into her life at a time when she needed rest to focus on her new baby.

No other participants were able to access NYC family paid leave after giving birth, which is provided to people who have worked for 20 or more hours a week for at least six months. In a few cases (*n* = 3), women had moved to NYC from other states during their pregnancy to seek family support and had not worked in the city, making them ineligible. But even the few that knew they were entitled struggled to make the company comply with the law. R.R., who had been working full time at a food chain for 2 years before giving birth, went back to work six weeks after, explaining: *“I went back to work but they (company) have not paid me yet for maternity leave, they don’t want to do the paperwork, and I am like forcing them*.”

In sum, the hardships experienced during pregnancy, limited unemployment and paid leave benefits that were not designed to protect women with unstable jobs, and potentially non-compliant employers, all pushed women into extremely precarious situations, including homelessness. In these circumstances, women described seeking support from their networks, the shelter system, and public assistance programs. After A.A. resigned from her job due to health complications, she moved to NYC to seek her family’s support. Her family, however, was facing their own hardships. AA then heard that she could get support at a shelter, where she was still living at the time of the interview and trying to figure out how to move on. As she described: “*I’ve never been so poor in my life. Poor. Have no money. …I didn’t think all of this would happen.”* The moment participants experienced these hardships was the time when they needed to navigate public assistance programs to find a way to get back on their feet.

#### Relying on Food Assistance and Informal Supports amid Scarcity

All women, regardless of their living and job conditions after birth, experienced food assistance programs as essential. All participants received food assistance after giving birth and at the time of the interview, only one was not using it anymore. Participants discussed how receiving WIC helped them pay for the child’s food and formula: *“WIC helps a lot, especially with baby food, like solids and stuff like that.” (H.H.)* WIC was widely received, and it was easy for participants to obtain and retain. Even when participants experienced “*being cut”* from other assistance (i.e cash) as they started working more hours, they did not lose food assistance entirely as B.B. described:

“Once they (public assistance office) saw that I was working — and they didn’t care if I was making $215 a week — It was automatically no this, no that, no Medicaid for you, you won’t — you’re not getting any more cash, but you could receive food stamps. But I went from $353 to $179 in stamps a month”.

However, although SNAP and WIC helped participants to cover their food needs, these benefits were most often not enough by the end of the month, especially after experiencing reductions in monthly benefits. As P.P. described it: *“Usually, by the end of the month, that’s when - before I get my food stamps, that’s when food runs out*.” This was accentuated for women not working and those either not receiving cash assistance or receiving small amounts of cash such as O.O. who had three children and was living at a shelter for victims of domestic violence *“It’s difficult when you get $250 every two weeks* (cash assistance). *I spend a good $60 on diapers. Then, when we run out of food stamps because my kids eat, I just have to buy her* (daughter) *snacks to keep her going”*. Not having enough by the end of the months also led some women to stretch the food they bought using SNAP: *“when I lost my job what I did was stretching it (*SNAP*). If there was no meat, we would eat eggs. I would buy one pack of corn flakes and not leave it around. I measured out what I would give them (*children*)” (L.L.)* or buy cheaper and less nutritious food. While food assistance was critical, mothers who experienced periods of no or very low incomes (making $100 per week) described how they had to rely on their families to cover other basic household expenses, baby items, cleaning necessities, or cell phone bills. “*My aunt, she pays my cell phone bill for me. At the end of the month, she’ll give me some money, and that’ll help towards all the other stuff that I need.” (J.J.)*

Participants also described the role of community resources to cover such expenses. For example, D.D. was getting help from the shelter with diapers, formula and clothes, while other mothers, such as G.G., would cut down on their expenses to cover indispensable baby items. *“There was one week, a few months ago, where I had enough money left over for toothpaste for myself”*. The problem of covering essentials was accentuated for undocumented immigrant mothers (*n* = 2), who were not eligible for SNAP and thus had to use any income they had for food. One such participant, who was diabetic and did not have enough resources to buy food consistently, told us: *“I am not eating well. What they (*WIC*) give me is not enough” (I.I.)* She was making $150 per week babysitting, living with a woman from her church, and receiving only WIC. Her experience highlighted the added problems for undocumented migrants. In contrast, the few mothers that were receiving disability income and housing assistance or that at some point after giving birth managed to work (part or full-time) and obtain childcare support (either by public assistance or family) described few difficulties covering their family’s food needs and baby expenses.

#### Waiting for Limited Affordable Housing: ‘Life on Hold’

Having a safe place to live was the other main priority for women and their babies “*I just want to have my own place for me and my child*”. Nine out of twenty women were living in shelters at the time of the interview, and two more had been in a shelter at some point before the interview. Living in a shelter permeated the women’s daily lives and worries. While the shelters provided a safe place, they also created stress and a sense of ambivalence. As one mother reported, “*I don’t want to stay in the shelter, but for now it feels like home*” while at the same time “*not having family around, not having friends. It takes a toll on you.”* Participants described shelters as a temporary solution, and the quality of their experiences depended on how much support they received at the shelter. While some women received a lot of support, for example in applying for benefits and obtaining baby items, others did not “*they gave you the crib, but they don’t help you. I asked my caseworker “where can I get baby food supplies?” she told me to google it. I would do four different housing specialists in a year. It was nerve-wracking.” (A.A.)”*

Despite the limited availability of public rental assistance, families living in shelters are supposed to have priority to access housing assistance. Across the nine participants who lived in shelters, six got a housing voucher and four of these six were using it or moving out soon. Two participants, who had been waiting for housing assistance for years, described how ‘*everything sped up’* when they entered the shelter. As J.J. described it: “*I was surprised when I got the housing specialist. She helped me with the application and I kept calling every week for an interview. I was surprised I got an interview with housing soon after I got here.”* With the support within the shelter and her persistence with the housing department, she finally got a voucher and moved to a small apartment where she paid $300 a month in rent that she covered using a portion of her disability assistance, which she received due to mental health issues. However, other mothers experienced the wait for a housing voucher as long and stressful. Participants described how they needed to show proof of stable income in order to access housing assistance. That in itself delayed the process, particularly when mothers struggled to secure a stable job.

“You have to work 30 h a week for a month straight before you can qualify for a voucher (housing). But right now I am not working because my job finished. It was temporary. I was a flu program coordinator. It used to be reasonable hours but I could not get to 30 h”. (D.D.)

Participants that were receiving disability benefits explained how having an income facilitated the process of accessing housing assistance. *“The guy* (housing specialist) *said – they* (housing office) *are like, food and shelter on the application… someone just on welfare. They don’t want that. If I didn’t have an income, I would be right back in shelter.” (J.J.)*. However, despite having an income was described as necessary to access rental assistance, one mother described how changes in income could affect her ability to retain the voucher that she much needed, which created an additional mental burden. B.B. described “*They* (welfare) *told me, if you go up on your days (*from part-time work to full-time*), you are not entitled to your voucher, which means that the apartment you just got, you have to pay out of pocket*.” She was making $115 a week and contributing to her rent, but was worried about not being able to cover her expenses if she started to work full time. These experiences reflect how burdensome and confusing navigating the process of accessing and keeping a housing voucher was for mothers. Not being able to secure an income combined with the lack of support to apply for assistance in some shelters brought a lot of uncertainty, affecting the well-being of participants and putting their lives on hold.

“It would be much better if I was able to have Section 8 and find a place where my kids could be free. Here (the shelter) it feels like a work-release prison. All your rights are stripped from you. I think mine and her (daughter) attachment would be a little bit better (if not here)’… I wanna go back to school, and I would be willing to work. Because I have to do both. I have to get out of this. I am at a standstill right now. I am in a funk. Bein’ here, I don’t wanna do nothing, especially livin’ in this neighbourhood, is just horrible to me. I feel once I leave, everything will be different.” (J.J.)

Most mothers navigating shelters and housing assistance programs reported anxiety, exhaustion, and even symptoms of depression as a result, as A.A. described: *“my pregnancy was terrible, I was really depressed. I went down a little once I had him. I was still depressed because I was in the shelter”*.

When women managed to access housing assistance, a new set of issues emerged. Participants’ challenges with the voucher process highlighted critical implementation gaps. For example, two participants described how property owners often refused to take vouchers. I.I. lost her voucher after not being able to use it, and at the time of the interview she was moving to New Jersey with the hope that it would be easier: “*They don’t want to accept it here. I’ve been looking for three months. They say that if I don’t have credit — income tax — they won’t rent it”*. I was a victim of domestic violence, only spoke Spanish, had been in and out of shelters for two years, and could not find a stable job. Not being able to use her voucher put her in an even more vulnerable position.

Participants that managed to use their vouchers described housing options that were limited given NYC’s high rental rates, which lead to having to move into more resource-deprived areas. Feeling “*lucky*” to finally get a Section 8 voucher, B.B, described how she had to take an apartment in an unsafe area:

“The vouchers are $1268. That does not even cover a studio in New York City. So finding a place that is willing to take you with a voucher is hard on itself…[When are you moving?]. My move out date is November 2^nd^, that is when my six months (at the shelter) is up. So for me, it was the first apartment I saw, I took it, in (name of the neighborhood). It’s in a drug-infested area, but I have no choice but to take it because I have to be out (of the shelter system).”

The experiences of these women suggest that while the system tries to provide housing assistance to the most vulnerable women that enter the NYC shelter system, larger systemic factors may impede housing assistance from working as it should. For the rest of the participants, the most common way of finding affordable housing was by living with family members. However, having insufficient income to afford an apartment for themselves and their children brought feelings of low agency and stress for numerous participants as H.H. described:

“Right now I can’t save. I am just living off what I make. I try, but when I do save I have to throw it towards a bill, not towards moving. My goal is to get a second job and move forward with my life. I want my situation to be different… I want my space, yes. Yes. It gets frustrating, and then that leads me to not checking up on my friends, not being a good friend. It just makes me not want to reach out to anyone, because I’m in this bubble of stress.”

Uncertainty about when they would have enough income to become independent made it hard to plan. While participants living with their families accepted that this was the best solution for the time being, and expressed gratitude to have their family support, they also described how having to depend on their families and share small spaces made them feel like “*I don’t want to be a burden”* and caused tension in their relationships. E.E. described how, after fighting with her mother and brother, she thought about leaving. However, she could not afford a place by herself and she did not want to enter a shelter: “*I am scared to live by myself, how am I gonna cover the rent when my job is not stable? When it is busy* (salon) *I get all this cash and tips, but when is slow, I only may get two clients”*. The dilemma between staying with family when conflicts arise or moving into a shelter showed the complex tradeoffs that mothers faced when trying to provide necessities like housing for their new babies as S.S., who lived with her abusive mother put it:

“I just had a baby, so do I really wanna walk into a dirty shelter with a brand-new baby? No. I did not feel like that then, and I still don’t feel like that now, so it just bothered me. I was like, “Shelter’s not an option. I’m not goin’ through it.” People will be like, “Why didn’t you, you’ll get your apartment in a year,” and I’m like, “I can’t.” I just couldn’t. I can’t find it in me. It’s just the whole process of it, too. I just can’t.”

Entering the shelter system was described as a way of getting housing assistance. However, it took an emotional toll that some women tried to avoid.

### Finding Pathways Towards Stability After the Baby’s Birth

For most women, except for those on disability, their priority while finding stable housing was to secure *any* job, as a pathway to have a more financially stable situation, and provide for their children. Nine of the participants were working at the time of the interview (only 2 full time), and four were looking for a job. Most participants described how they had to prioritize finding a job over continuing their education despite their desire to continue their studies in order to secure a better job for themselves and a better future for their children. A.A., who was in a shelter, described *“I got into nursing school, but I can’t go because I don’t have anywhere to live. Not even that, I don’t have nobody to watch him* (infant) *if I need time to study, or watch him in the morning…I wish I had the supports to where I could have went to school and started my career. That way we don’t have to go through this anymore*.” As A.A. experienced, more often than not, women had to put their educational desires on hold, with no supports to pursue them and pressure to find a job to both secure an income and access housing and childcare assistance. Only three participants managed to engage in educational activities, primarily because they received support from their family or caseworkers. In contrast, R.R. did not return to college after giving birth and found a full-time job while the baby’s paternal grandmother took care of the baby. She was receiving assistance for college but she needed to work, pay for NYC public housing and be with her baby.

“I was planning to go back to school. Now I feel I don’t have, not the desire, because I do want to go, but I don’t have the time. I work Monday to Friday and then come home with the baby. I could do online for two days but I don’t know if the government would pay for student assistance for a private college. Because I can’t miss work, I haven’t been able to go and get the information. I feel my time is running down. First I have to see how the daycare is going to look like and like my schedule. I have to learn how to balance everything together and see if everything connects.”

R.R. knew where to go to obtain child care and other resources, but was uncertain of the outcome, so it was hard for her to plan how to both work and go back to school. This struggle was common for mothers that describe how they wanted to find more stable jobs or study to access well-paid jobs and yet how they did not have the time, energy and resources. However, participants recognize how that was the pathway towards both financial and emotional stability for themselves and their babies “*I wanna be happy for my baby to be happy. I can easily get a makeup job somewhere* (what she was doing), *but I don’t want to do that. I’m trying to figure out exactly what I can do where I know I’ll be happy enough to stay and save.”(*H.H.)

One aspect that compromised women’s ability to find their pathway towards financial stability was access to childcare. At the time of the interviews, when children were almost 11 months, only six out of 20 mothers had access to public benefits that covered childcare and five women who were working were relying on their families to care for their children. When women had family members that could take care of their children, they chose that option first, as it was often convenient, flexible, and they knew them already. As children got older, mothers began considering other childcare options to provide socialization opportunities to their children and have a more structured form of care. For women in shelters, or with no family members that could take care of their babies, finding subsidized childcare was the only option to be able to find a job or go back to work, to pursue more stability.

Mothers trying to access subsidized childcare before having a stable job described a range of challenges to accessing such supports, such as work requirements. Mothers saw the need to have childcare as a prerequisite for applying and getting a job, however, this was not what they experienced when applying for a childcare voucher. As M.M. described it: *“I had a voucher that corresponded to my housing support, but they told me I could only use it once I was working. How was I supposed to look for jobs without daycare?”* Participants reported wanting to go back to work, but such rigid conditions for receiving assistance did not facilitate the process. Similarly, G.G. was told that once she gave her information to get a childcare voucher she should have to attend a ‘back-to-work’ program within three days, but she said *“I didn’t have anyone to watch the baby*.” While she believed that eventually, she would get the childcare voucher, such rigid implementation made the process burdensome and complicated, adding another layer of stress to her life. Such requirements were particularly complicated for women that lacked the family support to care temporally for their children while looking for jobs.

Having fluctuating paychecks and transitioning off of cash assistance were also described as barriers to securing stable childcare assistance. B.B., who was living in a shelter due to domestic violence and had recently stopped receiving TANF, explained her struggles: “*I feel like in a shelter with a baby, I should qualify for daycare. They were covering daycare, and then they stopped it because they said I work* (too much)*, and they do not seem to understand that daycare is a lot of money*.” Such issues to obtain and keep a voucher left some participants struggling to find childcare and relying on informal care options while trying to become financially stable. As E.E., pointed out: “*They literally do not care if I show them the different pay stubs, they are ‘no, this is what you are making now so you can’t have it* (SNAP and a childcare voucher*).”* E.E., who lost her benefits after travelling to her country to see her family could not obtain childcare assistance again. She had changing schedules and fluctuating income and saw her benefits reduced to match her highest monthly paycheck rather than her lived reality over months. These issues fueled worry about service discontinuities, adding an extra layer of financial burden.

In addition, most participants navigated the process of finding childcare alone and described having to deal with limited options in their neighborhoods, centers not taking their vouchers, or centers refusing to have children that showed challenging behaviors (i.e., “*crying too much”*). These barriers affected participants’ capacity to find work but also increased their mental burden.

“I have to look for the baby’s daycare before I can go to work. It’s impossible. Every daycare that I’m running into is (for children) two to five (years old), they don’t wanna take him cuz he’s one year; and when I finally do find the age-appropriate one, they don’t take the voucher. So I am stuck. And I don’t know a really good person to take care of him.” (C.C.)

This conundrum was accentuated for single mothers with limited support from their families. With no references or recommendations, trusting people they did not know to care for their child was seen as an additional challenge. Mothers longed for guidance to facilitate the process of finding childcare and using childcare subsidies.

“Welfare’s willin’ to pay either the daycare or even a person, but I don’t even have a person that I trust, that I know that’s gonna be like, “This is your job.” I don’t wanna leave my baby to somebody that’s gonna do half of a good job. I don’t really know a good person…so it’s hard. I’ve literally been, still, no job, nothing, in the same spot, because it’s like, “Where to go? What to do,” and then I gotta look for all the resources.—I hope someone would tell me “Here, there is a childcare here and you can use the voucher” so that I can go back to work” (G.G.)

In contrast, four women in our sample, all of whom happened to work, described better access to childcare assistance and good experiences finding childcare centers near them, suggesting easier access for already “working mothers” and how differences across neighborhoods in childcare options (availability, quality, acceptance of vouchers) intersected with families’ ability to access and use childcare assistance.

### Making It Work: Efforts to Look Forward

Despite the barriers and challenges described above, virtually all the mothers in our sample reported putting their child’s needs first and finding strength in their children. Many went to great lengths to pull together resources from community organizations, family, and public assistance programs to cover their children’s needs. When L.L became pregnant, single, and jobless with three children she mobilized all available supports

“I started looking for information. Who could help me, who couldn’t, who could I talk to. I looked for a social worker. A hospital helped me find places where I could get diapers. I started to prepare financially. Emotionally, I talked to my kids, to my friends. They supported me. They gave me support. I told them what was going on and all of those things, and I got emotional and financial support.”

All participants described how they made sure their child’s basic needs, such as food and having a safe place to live, were covered first and foremost, even if their financial situation would not allow them to buy toys or books for their child. F.F. reported: “*My main thing is her food and rent. Whenever I do have $10, I’ll be: ‘Okay, she needs this.”* Beyond material goods, participants described how their priority was to be a caring mother despite all the stress they felt given their limited financial resources. As H.H. put it: *“As long as she’s getting read, she’s eating, her diaper’s changed, food in her belly, clothes on her back, she’s playing, she’s a happy baby — that’s all that matters.”* The child’s well-being was top of mind constantly for all participants, as all their actions were driven by their desire to provide a better future for their children despite the challenges they faced as they tried to move out of poverty. When children’s needs came first, participants often neglected their own needs: “*It’s never like: ‘Okay, I need this.’ It’s more like: ‘She needs it.” (*F.F.), sometimes to the detriment of their well-being:

“Sometimes it’s just hard for me to focus. When I become stressed, it’s just hard to focus. The main thing I do focus on is my child. I make sure she’s eating, and I make sure she’s happy. I make sure I’m doing what I’m supposed to do as a parent…I keep forgetting, like, ‘Take care of yourself too.” (H.H)

The stress they experienced - from having to navigate unstable jobs, unstable housing, and the limited short-term possibilities to change their situation – made it difficult for them to care about their well-being. Nonetheless, across the interviews, some women described how their future started to look a bit better. In paying close attention to their stories, we observed a common pattern. Mothers described individualized support that helped them navigate and coordinate all the public assistance programs and community supports that were available to them in the best possible way. One mother described how, through a social worker at a non-profit organization (not Room to Grow), she found free childcare, which gave her the flexibility that the childcare voucher, with strict work requirements, had not given her. That allowed her to have the time she needed to finally finding a stable job. Another mother described receiving job training through public assistance that helped her prepare for a more stable job in the future. She described: *“I like the job specialist. I don’t have to work now because I have a disability but I feel I want to work in security. The specialist helped me take the city exam and I passed.”* Such support benefited mothers’ capacity to think long term and find the right job without undue pressure. Mothers reiterated the value of such supports across other domains as well, such as finding stable housing and childcare and continuing with educational pursuits. N.N. described the pivotal role that her social worker at Room to Grow played in helping her to utilize her voucher before losing it: “*They played a big part. It was really hard to look for an apartment. Had the voucher for 6 months, couldn’t find anything - and she found me an apartment that would take it!”*

Similarly, the three participants that described being able to enroll in educational programs also received significant support from their close family or a social worker. D.D. entered a homeless shelter with her two children when she was unable to afford an apartment with the income from her job at a dollar store. Once in the shelter, she received support from her caseworker to apply for cash assistance, childcare, and student financial aid to finish her associate degree. Also in getting baby items such as diapers and wipes. She described: *“I’ve been getting a lot of help from my case worker. I have a wonderful caseworker. I speak to her and she helps me figure it out.”* D.D. not only received support to cover her material needs and apply for benefits but also support in using them: *“My caseworker is the one who advised me about one daycare. I went down, saw how it was set up, how the caretakers took care of children…But it was really hard…putting your children with people you don’t know*.” Despite she struggled at first, this support allowed her to take a part-time job and put a lot of effort into finishing her education. D.D.’s case demonstrates that when assistance is coordinated, comprehensive, and flexible, mothers could focus their energy on educational opportunities that could potentially lead to better jobs and greater stability in the future.

Similarly, N.N. found practical and emotional support in her social worker at Room to Grow to finish her GED while receiving cash assistance and living at the shelter. “*she pushes me to go harder, she gave me all the information, to go and do (GED)…I would have never gotten my GED. I would had to go and get a full-time job. I would not had the time to do what I had to do for myself, to better myself.”* Recipients of temporary cash assistance in NYC are required to engage in work and/or educational activities, yet the focus continues to be on finding a job. N.N. received all the information to enroll in a free GED program that would allow her to keep the cash assistance and childcare she needed to finish her education and plan her long-term goals to have a more stable life “*Next is I wanna go pick up a trade for Certified Nursing Assistant. From there, I wanna get into this CAN-get more stable than what I am now, and I am planning on going to college.”*

## Discussion

This study set out to explore how predominantly Black and Latinx mothers in NYC experience living in or near poverty in the time surrounding pregnancy and the first year of their child’s life while navigating the “patchwork” of safety net supports to meet their family’s needs. Research suggests that public assistance programs have the potential to reduce poverty and its adverse consequences for maternal health and child development (Bartfeld et al., 2015; Sherman & Trisi, 2015; Wimer et al., 2016). Qualitative results from this study confirm the perceived benefits of in-kind public assistance programs. All mothers in this study were engaged with some type of in-kinds public program. Mothers described how food assistance covered the basic food needs of children (Bruening et al., 2017), and became often a “life saver” (Robbins, Ettinger, Keefe, Riley, and Surkan, 2017), despite running short by the end of the month (Nieves et al., 2021). Likewise, when mothers managed to obtain and use housing or childcare assistance, they were able to allocate their low incomes for other daily expenses (e.g., diapers, household expenses), which reduced financial pressure. Nonetheless, results also highlighted mothers’ struggles to access rationed supports (housing and childcare), often due to work requirements, and to use them with limited coordinated assistance, lack of information, or limited available options. Such challenges ‘pushed’ women to focus on short-term solutions to cover their pressing immediate needs - housing, work, and childcare - leaving little room to engage in long term strategies, such as continuing with their education (Freeman, 2017) or building savings, thus adding mental burden and stress into their lives when their babies needed them the most.

Our results expand prior qualitative research among mothers living in or near poverty by highlighting some crucial aspects relevant for women around the time of pregnancy and after birth. This study shows that while states such as New York have implemented family-friendly policies, such as paid family and sick leave, women with unstable jobs and experiences of poor health, stress, and domestic violence, may not benefit from these policies, which pushes them to very precarious situations, even if small amounts of cash (TANF) are provided. Indeed, results showed how single mothers that lacked strong family support had to rely on the shelter system to stabilize their economic and family situations. Pregnant women experiencing homelessness are at increased risk for mental health problems and prenatal and birth health complications (Clark et al., 2019), which then require more support from public assistance programs. The shelter system provided very heterogeneous experiences to mothers, with some experiencing very little support and others experiencing coordinated services that facilitated access to cash, housing and childcare assistance, therapy and free baby items. Thus, women’s access to timely assistance may depend on how much information and resources they receive within the shelter system. When mothers cannot access timely and affordable housing after giving birth, they experience their lives “on hold”, taxing their mental well-being and complicating their ability to find pathways towards financial and emotional stability (Aratani et al., 2019; Bovell-Ammon et al., 2021). Access and use of public assistance (i.e. housing assistance) after entering the shelter system can reduce subsequent homelessness (Fowler et al., 2019)), therefore, we must further explore what factors create these differences in quality and service delivery within the shelter system.

Our findings also raise questions about potential miscommunication or lack of information about requirements for accessing childcare and housing vouchers. Mothers in our study voiced how they were required to “work first” or have an income to access housing and childcare vouchers and how they experienced these criteria as very inflexible, contributing to their stress and mental burden, particularly when they lacked support from social networks. Freeman and Dodson (2020) found similar results across different states. However, despite the current safety net focus on incentivizing work, NY state regulations should allow mothers looking for a job to access subsidized childcare. Likewise, although work requirements in housing assistance programs are becoming more common (Frescoln et al., 2018), lack of income should not be a criterion to be excluded from housing assistance. Prior work has suggested that the complexity of the safety net, allows caseworkers to use their discretion to decide who “deserves” to be provided with limited resources and when (Taylor, 2014). Our study did not utilize data on the perspectives of caseworkers, however, the conundrum we described suggest once again that caseworkers, may play a large role in facilitating or hindering mothers’ timely access to safety net programs. Findings suggested that support from caseworkers facilitated actual use of assistance once it was granted Overall, more research is needed to understand how public assistance programs that provide in-kind or cash support can be implemented as intended and produce the desired outcomes by using the guidance and support of caseworkers, within and outside the welfare system.

Results also demonstrated how mothers often adapt and cope with the frustration and stress of living with very low incomes by putting all their energy into their babies. Prior research has shown that motherhood among young parents living in low-income households can create a sense of control and provide direction and purpose in otherwise chaotic life circumstances (Solivan et al., 2015). However, our findings also point out that when mothers do not have the resources that enable them to focus on their long-term goals, it can lead to stress and worry affecting their mental wellbeing and workforce participation (Bush et al., 2017).

### Implications for Practice and Policy

Our presentation of these day-to-day experiences is not intended to minimize the need for existing benefits, nor to imply that public assistance is not effective in reducing poverty. Rather, our participants’ experiences offer meaningful data that can contribute to current debates on how to improve the safety net for families with infants.

First, results show a need for more income support for pregnant women with temporal, unstable and low-income jobs that are not protected by tax programs and family-friendly policies, such as paid leave. In our study, even mothers receiving small amounts of cash assistance barely made it to cover their family needs. The current government COVID-19 stimulus, under the American Rescue Plan Act (ARPA), has enhanced the child tax credit in value and has extended it to cover children whose families had the lowest incomes by divorcing the credit from the earnings requirement (Jaffe, 2021). Under these emergency funds, families with children under six years old received monthly payments for a total annual value of $3,600 per child until December 2021. Our results argue for a continuation of such policies that expand coverage to provide a basic floor of cash income support (Collyer et al., 2020) and avoid living with no income and experiences of “*I’ve never been so poor in my life.”* around the time of giving birth.

Second, paid family and sick leave programs with better coverage for mothers who experience job instability could prevent some of the cascading effects of health problems and interpersonal conflicts during pregnancy that led mothers in this study to enter the shelter system or depend on their families. Research has shown that longer parental leave and a higher allowance facilitate employment and are associated with lower poverty among single mothers (Maldonado & Nieuwenhuis, 2015), thus showing the potential of making such changes.

Third, there is a need for more robust housing policies that protect pregnant women from becoming homeless or staying long periods within the shelter system. Some scholars and policy experts have argued for providing universal housing assistance for extremely low-income households (Desmond & Gershenson, 2016; Galvez et al., 2017) and expanding the housing choice voucher program. The highly rationed nature of housing assistance makes affordability extraordinarily difficult when mothers experience a period of very low income, for example after losing a job during pregnancy. This adds unnecessary stressors to mothers and children during one of the most critical periods of vulnerability across the life course.

Fourth, public assistance should facilitate access to skills-building programs and higher education to improve the long-term job opportunities for single mothers, which would also allow them to benefit from current work-based policies, such as tax credits or sick leave (Hernandez & Napierala, 2014; Sommer et al., 2012). This would mean that mothers with low incomes - wanting to finish or pursue higher education - could do so with the support of cash assistance to cover their family’s needs (Dodson & Deprez, 2019). NYC state policies accept attending school as a reason to access subsidized childcare and cash assistance, however, mothers in this study experienced the pressure of the current work-based safety net to work over continuing their studies. The success of two participants in continuing their education suggests that caseworkers may have a crucial role to support mothers in finding feasible opportunities and accessing the public assistance they need to do so (Freeman, 2017).

Last, access to subsidized childcare *first* would perhaps reduce the stress that single mothers experience and enhance their ability to focus on finding a job or continuing their education after giving birth. Despite NY State considering looking for a job as a reason for subsidized childcare, this may not be always communicated by frontline workers when there is limited availability of resources (Hagen & Owens-Manley, 2002). Thus, there is a need to monitor more closely how childcare assistance is implemented and delivered and ensure that information about entitlements and regulations, and available high-quality childcare options is clearly provided to facilitate access and use of childcare subsidies. Hotz and Wiswall (2019) argued for a childcare subsidy expansion paired with an expansion of state and local platforms that inform parents about the quality of childcare options. This may be even more important for single mothers overloaded by the pressure of having to provide for their babies while living with low incomes.

Our research demonstrates the importance of listening to mothers’ experiences with navigating the full patchwork of public programs aimed at assisting families and children experiencing periods of low incomes. These experiences help us to understand how limited resources, lack of coordination, and lack of clarity about work requirements affect mothers who are trying to raise a newborn in a time of economic scarcity in their lives. We know that implementation of programs matter (Bertram et al., 2015) and that there are a set of necessary conditions - at the interpersonal, organizational, and systems levels - to make the patchwork of public assistance more coordinated and effective during the time around pregnancy and infancy. But the perspectives of mothers highlighted here point to the need for more innovative solutions to address benefit cliffs and further coordinate the needs of families living in poverty, front line social service workers, and legislators. Recently, the “Whole Family Approach to Jobs” initiative by the six New England states created teams of parents living with low incomes, legislators, executive branch leaders, businesses, and community-based organizations to address benefits cliffs and discuss systems reforms (NCSL, 2019). This partnership led states to revise and increase certain benefit rules (e.g., in SNAP and TANF) to make them more flexible for parents with a newborn. The initiative also developed interagency plans to coordinate service delivery, including tools to advise families about benefit cliffs and start designing two-generation approaches to improving support for parents and children. Initiatives like this one demonstrate the importance of moving beyond improving the safety net *for* families to making changes in the safety net *with* families living in poverty, thus ensuring more active and authentic participation in decision making.

### Limitations

This study does entail some limitations. First, while all mothers in the sample had low incomes, our sample was heterogeneous in terms of individual experiences. For example, some participants had experienced domestic violence, some lived in shelters, and some were teenage mothers while others were in their thirties and had other children. Such different circumstances and experiences could have influenced how participants perceived and navigated public assistance programs. Nonetheless, despite the small sample, we aimed to capture differences in experiences that emerged in the participants’ responses. Second, our data reflected participants’ subjective experiences and therefore our study was not designed to question whether antipoverty programs reduced poverty, improved health, or improve mothers’ situations. Rather, our study aimed to voice the experiences of mothers with low incomes who were engaged with public assistance during pregnancy and the first year of their babies’ life. In addition, our interviews were not designed to inquire into all the details of each program that women engaged with. Instead, we aimed to provide a broad picture of mothers’ experiences navigating assistance while trying to meet their family’s needs. Third, our study entailed a small sample and focused on the experiences of minority mothers from a single urban area with low household incomes. Therefore, findings should not be expected to generalize to suburban or rural populations without further exploration. In addition, we did not consider the role that Medicaid (health insurance coverage) plays in the life of participants. Finally, half of the study participants were receiving services at Room to Grow when they were interviewed. Experiences with the Room to Grow program could have influenced participants’ perception of their need for public assistance given that some sampled mothers received material goods for the baby (clothes, toys, books). Nonetheless, Room to Grow did not provide assistance that was offered through in-kind or cash assistance programs. Findings did suggest that personalized support from social workers and caseworkers (at Room to Grow and outside Room to Grow) facilitated in a few cases the use of public assistance (for example using vouchers). Studying the impact of Room to Grow was outside the scope of this study, however, the evaluation of the intervention will investigate in-depth whether Room to Grow can facilitate access and use of public assistance and other community resources across the three-year program intervention.

## Conclusion

In conclusion, the voices and experiences of the mothers in this study call for a comprehensive, critical review of current work-based public assistance programs and the policy details that dictate how they are implemented on the ground during pregnancy and after giving birth. Policies must do more to ensure that mothers, particularly heads of households, are healthy and financially stable in the long term. Future design and implementation of anti-poverty programs should consider ways to not only expand coverage for women experiencing poverty in the time surrounding pregnancy and the first year of life but also to facilitate longer-term solutions for mobility out of poverty, such as stable, accessible housing and childcare assistance, a less steep cliff of benefit drop-off that can prolong benefit periods, and improved access to subsidized or free higher education as a road to equitable workforce participation.

## Supplementary Information

Supplemental material 15Feb22
