# Supplementary material for: Poverty after Birth: How Mothers Experience and Navigate U.S. Safety Net Programs to Address Family Needs
Source: J Child Fam Stud. 2022 May 6;31(8):2248–65. doi: 10.1007/s10826-022-02322-0 (PMC9073812; doi:10.1007/s10826-022-02322-0)
Supplement: Supplementary file 1 — Supplemental material 15Feb22 [file 10826_2022_2322_MOESM1_ESM.docx]

**Supplemental materials**

| **Table S1. Summary of Public Assistance Programs in New York** | | | |
| --- | --- | --- | --- |
| **Program name** | **Benefits/Support Provided** | **Eligibility criteria as of 2019** | **Link to Law/Policy** |
| **Income support** |  |  |  |
| Temporary Assistance for Needy Families (TANF)-Family Assistance | (Federal/state-run) Up to 60 months (lifetime) of federally funded cash assistance for food, utilities and housing expenses. The national average is $445 per month. TANF recipients should directly qualify for subsidized childcare when they start working | -Pregnant women or responsible of a child under 19 with very low income (i.e: in New York State, the income limit is approx. $800/month for a family of three) or homeless | <https://www.acf.hhs.gov/ofa/programs/tanf/laws-regulations> |
|  |  | -States must engage in work activities at least 50% percent of all families with a work-eligible individual and 90% of two-parent families with two work-eligible individuals. Single parents must engage in work activities for at least 30 hours/week. Women are exempt from work requirements for the first 3 months of a babies life |  |
| Supplemental Security Income (SSI) | (Federal) Disability benefit of maximum $2,000 for an individual. The program also offers services to help them become employed: vocational rehabilitation, training, referrals, job coaching, counselling and placement services. | - 65 or older, blind or disabled and have limited or no income | <https://www.ssa.gov/ssi/ssi-law-regs.htm> |
|  |  | -An adult 18 - 64 who is disabled and has limited or no income and therefore can't apply for SSDI |  |
| Social Security Disability Insurance (SSDI) | (Federal) Disability benefit (estimated average monthly SSDI benefit is $1,277). The program also offers services to help them become employed. Social Security allows up to 45 hours of work per month if you're self-employed and on SSDI. That comes out to around 10 hours per week. | - People with a severe physical and mental impairment that prevents them from working and that is expected to last at least 12 months | <https://www.ssa.gov/pubs/EN-05-10029.pdf> |
|  |  | - Must have to have contributed to the social security system (taxes) for a certain amount of time. |  |
| Child Tax Credit (CTC) | (Federal) tax refund. The maximum amount per qualifying child in 2018 was $2,000 per year. Up to $1,400 of that amount can be refundable for each qualifying child. | - Parents with children under 17  -Must have an earned income of more than $2,500. Earned income can be from wages, salary, tips, employer-based disability, self-employment income, military pay, or union strike benefits.  -Must have a social security number (SSN) or an Individual Taxpayer Identification Number (ITIN). | <https://www.ncsl.org/research/human-services/child-tax-credit-overview.aspx> |
| Family paid leave | (NY State) 60% of weekly salary up to 10 weeks (i.e to care for the baby) | - Full or part-time work for at least 6 months before taking leave | https://www1.nyc.gov/nycbusiness/description/paid-family-leave |
| **Nutrition** |  |  |  |
| Special Supplemental Nutrition Program for Women, Infants, and Children (WIC) | (Federal) Supplementary food and WIC card to shop for your family’s WIC foods at WIC-approved stores; Referrals to other programs and services (i.e. cash assistance); Breastfeeding support | - Pregnant and post-partum women with children up to age 5 have incomes below 185% of the poverty line. | <https://www.fns.usda.gov/wic/wic-laws-and-regulations> |
| Supplemental Nutrition Assistance Program (SNAP) | (Federal) near-Cash (credit card) to purchase food. Depends on household size: Maximum for a family of 2 is $459 x month. | - Gross monthly income below 130% of the poverty line ($39,756 annual income for a family of 4) and assets (bank accounts) must be less than $2,250. | <https://www.fns.usda.gov/resources?f%5B0%5D=program%3A2&f%5B1%5D=resource_type%3A17> |
| **Housing Assistance** |  |  |  |
| Section 8 | (Federal/ administered locally by public housing agencies (PHAs) Housing choice voucher program that supports payment towards paying for housing in the private market. Maximum housing assistance is the lesser of the payment standard minus 30% of the family's monthly adjusted income. | - Family's income may not exceed 50% of the median income for the county or metropolitan area in which the family chooses to live  - Victims of domestic violence and homeless have priority | <https://www.hud.gov/topics/housing_choice_voucher_program_section_8> |
| Public housing New York city housing authority | (NY State) It offers a rent subsidy in one of the near 180,000 apartments located around the five boroughs of New York City-owned and operated by NYCHA. Rent is based on 30% of the applicant household’s anticipated gross annual income Allow deductions of $480 for each dependent; $400 for an elder, or a person with a disability; and some medical deductions for households headed by an elderly person or person with disabilities. | - Family income may not exceed $95,450 annually for a family of four (200% above the poverty line)  - Victims of domestic violence and homeless have priority | <https://www1.nyc.gov/assets/nycha/downloads/pdf/NYCHA-Fact-Sheet_2021.pdf> |
|  |  |  |  |
| **Childcare in NY** |  |  |  |
| EarlyLearn | (NY State) Tuition subsidized spot at a government-funded child care center (family or centre-based childcare center contracted by the city of NY) | -EarlyLearn programs are available for infants and toddlers (ages six weeks to 2 years old). The family must demonstrate low-income (up to 200% State Income Standard) | <http://www.nysed.gov/early-learning/laws-and-regulations> |
|  |  | - Family must demonstrate a 'reason for care': work 20+ hours per week, be in an educational or vocational training program, have been looking for work for up to 6 months, live in temporary housing, attending services for domestic violence. Families receiving TANF have priority |  |
| Childcare voucher | Childcare voucher used to purchase care outside or within (EarlyLearn) the city’s contracted system of a childcare center | - Same as above. | <https://access.nyc.gov/programs/child-care-voucher/> |
| *Note.* Criteria eligibility are based on data from 2018-2019 when this study was conducted. Non-US citizens are eligible for childcare, WIC, and income support if they have satisfactory immigrant status. Undocumented migrants are excluded from all listed programs except WIC and childcare (if the child is born in the US). When programs are New York State-specific it is described. In NYC, the Department of Social Services (DSS), which includes the Human Resources Administration (HRA) and the Department of Homeless Services (DHS), administers most of the safety net programs, including Cash Assistance, SNAP and parts of Medicaid. The Department of Education (DOE), in partnership with the Administration for Children's Services (ACS) runs EarlyLearn NYC | | | |

| **Table S2: coding scheme** | | |
| --- | --- | --- |
| Themes | Sub-themes | Codes |
| **Experiencing cascade effects of hardships during pregnancy** | | |
|  | Interpersonal conflict accentuates emotional and financial instability | |
|  |  | No financially prepared for unplanned pregnancy |
|  |  | Father not involved lead to precarity |
|  |  | Domestic violence brings stress and health problems |
|  |  | Domestic violence forced to quit job |
|  | Pushed out from unstable jobs with no income | |
|  |  | vulnerable to lose jobs with health problems during pregnancy |
|  |  | Unstable jobs, no paid leave |
|  |  | incompliant employers with paid leave |
| **Relying on food issistance and informal supports amid scarcity** | | |
|  | Food assistance is essential but not enough | |
|  |  | Food assistance easy to access |
|  |  | WIC good help to cover baby food |
|  |  | SNAP runs out by the end of the month |
|  |  | Cutting down on food for oneself to stretch SNAP |
|  |  | Cutting down the variety of foods to stretch SNAP |
|  | Relying on informal support amid limited cash to cover personal and baby expenses | |
|  |  | Diapers are a large expense |
|  |  | Cash support (TANF) is not enough to cover basic expenses |
|  |  | Need to use diaper banks |
|  |  | Family helps pay for basic needs (cell phone, cleaning necessities) |
|  |  | Cutting down on mother's necessities |
|  |  | Finding baby items via thrifting, charity and baby showers |
|  |  | Room to grow helps with baby clothes and toys |
| **Waiting for limited affordable housing: 'life on hold'** | | |
|  | The shelter environment shapes access to assistance and well-being | |
|  |  | No income and no family support lead to enter the shelter |
|  |  | Domestic violence leads to entering a shelter |
|  |  | Long stay in shelter fueling stress and despair |
|  |  | Shelter accelerates housing vouchers |
|  |  | Rigid income requirements to get a voucher |
|  |  | Caseworker facilitate access and use of assistance |
|  | Challenges with access and use of public housing assistance | |
|  |  | Rigid income requirements to get a voucher |
|  |  | Voucher value forces to live in resource-deprived areas |
|  |  | Landlords refusing voucher |
|  |  | Hard to use a voucher with no help |
|  | Feeling stuck when there is no prospect of an independent life | |
|  |  | Gratitude for the family for providing basic housing |
|  |  | Limited privacy brings conflict |
|  |  | Better avoid entering a shelter |
| **Findings pathways towards stability after the baby's birth** | | |
|  | The work and education dilemma | |
|  |  | Pressure to secure an income to cover baby expenses |
|  |  | Educations will bring me further |
|  |  | No supports to study |
|  |  | Not sufficient income to live without public assistance |
|  |  | Finding a job to be self-sufficient |
|  | Childcare issues as a barrier to employment | |
|  |  | Convenient family support to care for infants |
|  |  | Navigating childcare system alone |
|  |  | Burden by pressure to find work first |
|  |  | Assistance available but hard to trust childcare providers with no references |
|  |  | No childcare available in the area |
|  |  | Experience with income cut-off and discontinuity of childcare assistance |
|  |  | easy access to subsidized childcare while working |
| **Making it work: efforts to look forward** | | |
|  | Putting the child first | |
|  |  | All for the baby |
|  |  | Baby gives me strength |
|  |  | After the baby, I am a better person |
|  | Agency mobilizing resources | |
|  |  | Go look for help once I was pregnant |
|  |  | Try to work more during pregnancy to save |
|  |  | Asking for help at the hospital |
|  |  | Reaching out to community organizations |
|  | Customized and flexible support helps navigate assistance | |
|  |  | Help to find an apartment to use a housing voucher |
|  |  | Recommendation of childcare build trust |
|  |  | Flexible free childcare via community agency |
|  |  | Help findings free GED program |
|  |  | Job specialists provide useful support |
|  |  | The caseworker provide emotional support and encouragement |

**Interview Guide**

**PREGNANCY**

1. What was your life like when you found out you were pregnant? How did you feel about it?

(Probe: work? Living conditions? Public Assistance programs)

1. As you got far along on your pregnancy, what kinds of preparations had you made for your child? What kind of supports you were receiving (Probe: Public Assistance). How those supports helped you?
2. Did you look for any support you thought you might need before your baby was born? If yes, tell me more about these supports? Why did you look for them?
3. Did someone or something help you get ready for your baby? How?
4. Was there anything that worried you? Why?

**BIRTH**

Now let’s talk about the end of your pregnancy:

1. How was your experience of having birth? How was the support and care you received?
2. How did you feel when your baby was born? And the weeks after? Why?
3. Did you receive any support then? If yes, how was the support you received? How did you feel about it?

**FINANCES**

1. What kind of finances do you have? How do you afford your and the baby’s needs?
2. What kind of Public Benefits do you receive? (Probe: cash assistance, food, housing, etc..)
3. What do you think about the supports you receive? How do you manage them? How do you access them? Are they enough? How do you use them?
4. Do you have any other support or resources to cover these expenses? How do you use them?

**SUPPORTS**

Now, I’ll ask you some questions about supports you may have received or looked for. This can include support from people, from agencies, organizations, Public Assistance:

1. Is there anybody that you rely on to raise your baby? Who have been the most helpful sources of help so far? (Probe: partner, friends, family, parents, organizations, etc.). Why?
2. Since your baby was born, were you offered any services for you as a parent or your baby? If not, did you look for any services for you and your baby?
3. How has your experience been? Tell me more about it. What has been helpful and what has not been helpful so far? Tell me about anything you have learned through your experiences with such supports.
4. Are there any kind of support you think would have been useful for you and your baby?
